# Abstract of the Proceedings of the Chicago Medical Society

**Published:** 1860-01

**Authors:** E. A. Steele

**Affiliations:** Sec’y pro tem.


### ABSTRACT OF PROCEEDINGS OF THE CHICAGO MEDICAL SOCIETY.

The Society met, pursuant to notice, in the College Hall, Dr. Waite in the chair. Present: Drs. Hollister, Isham, Wickersham, Smith, Jones, Barrows, Allport, Peake, Graham, Andrews, Steele, and Prof. Deville, with a number of the Medical Students of Lind University.

Dr. Davis being absent, Dr. Steele was appointed secretary

*pro tem.* The minutes of the last meeting were next read and approved.

Prof. Deville, having been proposed, was duly elected a fellow.

Dr. Steele, the essayist of the evening, read a paper on the pathological effects resulting from the ingestion of alcohol, demonstrating how the usual functions of the nervous system were increased or perverted without a corresponding excitation of the vital properties; and that these vital properties being lowered down, how the tone and strength of the system was impaired—the processes of the metamorphosis of the tissues, and the production of animal heat being retarded; and how, that alcohol by its peculiar affinity for the albuminous constituents, enters into combination with all the tissues, producing by its presence in the blood two distinct and separate pathological conditions, which give rise to what we are pleased to call delirium tremens. The one supervenes upon high stimulation in robust healthy constitutions, unaccustomed to regular indulgence; and either from the quantity ingested in a short space of time, or because the individual is more susceptible to its effects, we have direct irritation upon the stomach and brain producing a morbid susceptibility of the nervous system, with organic force sufficient to give "active manifestation to the symptoms." The second we have occurring in those who have had previous attacks, and are worn out through the influences that were shown to result from the secondary effects of the predisposing cause, producing all the effects of a perverted susceptibility, without organic force sufficient to give active manifestation to the symptoms, and the vital powers yield with a futile attempt to respond to the excited impression made upon them.

Concerning the treatment, the Doctor recognizes *three* general indications to be fulfilled.

1st. The depuration of the blood of the hydro carbonaceous material floating in the system. 2d. Allaying the consequent morbid susceptibility. And, 3d. Restoring the balance of nutrition by giving support to the organic functions.

The use of ardent spirits was deprecated in full terms in the sthenic form, as only serving to exasperate its tendency; and also in the asthenic, he considered that the general indications could be most readily fulfilled by withholding the accustomed stimulus. The other two indications could conjointly be the most successfully reached by recruiting the blood with rich pabulum, and by the exhibition of such remedies as are well-known to exert a quieting influence in overcoming the morbid susceptibility, and giving power and efficiency to the organic properties in restoring the secretions, and thereby depurating the blood and equalizing the circulation. He further considered, that opium was not the only remedy to fulfil the indication, but remarked, that the internal administration of chloroform combined with ti. opii. had been followed by the most desirable effects in several cases. It served to allay the morbid susceptibility by producing, as it were, anæsthesia of the sensory nerves, and exciting a paralizing influence on the muscular fibre, and that too without depressing the "organic functions;" differing in this respect from the ordinary sedatives, but acting seemingly as a powerful anodyne and mild stimulant.

The views set forth in the essay provoked an interesting discussion, which was participated in by Drs. Deville, Hollister, Isham, Graham, Smith, Waite, Andrews, Wickersham, Fisher, Steele, and Prof. Taylor.

The sanitary reports being called for, Dr. Wickersham, of the South Division, the only one of the Committee present, asked to be excused until the next evening.

The question, "What are the characteristics of the fevers prevalent in the city the present autumn?" was read, and the discussion opened by Dr. Waite. Dr. Wickersham in the chair. The disputant remarked that he had very little or no experience in the treatment of the fevers of the present autumn, in the city.

A motion was made by Dr. Isham, and carried, to defer the further discussion of the subject until the next evening.

Dr. Isham next exhibited to the Society a mass of fibrous tumors of the uterus, which he accompanied with a verbal history of the case, and description of the specimen, the outlines of which were as follows:

"The subject from whom this was taken some months ago, was 42 years old; had had one child, now living, 22 years of age. She always menstruated regularly up to death, and did so only two weeks previous to that event. She first discovered this tumor in the left iliac region 17 years ago, since which time it has regularly increased in size, and when I saw her, it filled the pelvis, extending above the umbilicus, and could be felt as a large irregular mass, which, floating in a serous effusion, enormously distending the abdominal walls, readily yielded to pressure and gave the feeling similar ballottement. There was great œdema of the lungs, accompanied by dyspnea from the upward pressure; also serous effusion, greatly distending the limbs, from pressure below. She was unable to sleep, except in the upright position, and under the influence of anodynes.

Being urgent for relief, and satisfied that she could not live another twelve hours without, as she said, I taped the abdomen, and drew off twelve quarts of clear serous fluid, and bandaged her, leaving her comfortable; she passed a good night, greatly relieved and refreshed thereby.

She lingered for nine days longer, when she died from syncope, whilst in the act of defecation at her bed-side, without any pain or struggle, so quietly that her son, who was in the room at the time, was unaware of the fact until he went to assist her back to her bed.

In removing the tumor at the autopsy, it was noticed that there were no adhesions, and was separated from its attachments without difficulty. There was some fluid in the abdomen, with occasional patches of recent lymph upon the peritoneum. There were also effusions in the pleura and pericardium, and œdema of the lungs. The weight of the mass is 12½ pounds, consisting of an infinite number of fibrous tumors, varying in size from a pea to that of a melon; whilst in the structure of the uterus and from its outside, quantities of these tumors are found existing as encysted or pedicellate; the internal or mucus surface of the uterus is quite healthy and normal in appearance. The largest of these tumors weighs about 6 pounds, and some of the encysted appear to be encased in a shell of calcareous matter, containing no bone, but porous like the pumice stone.

The Doctor also called the attention of the Society to the fact, that as this patient had only one child, so also on examination of the ovaries, which were natural, was to be found a well-marked corpus luteum, exhibiting all its characteristics in a marked degree. In other parts of the ovaries were to be seen graaffian vesicles in the different stages of development, one of which had apparently burst but a short time previous to the death of the patient.

This specimen is deposited in the Museum of Lind University, and was referred to a Committee, consisting of Drs. Deville, Isham, and Andrews, to report at some future meeting more freely upon its microscopic character, and the pathology of fibrous tumors.

Dr. Hollister also presented a very interesting specimen; showing almost an entire cast of the trachea, as far as the bifurcation, and extending into the small ramifications of the bronchia. It was hollow throughout, and it was evidently fibrous in its organization.

On motion, the Society requested Dr. Hollister to accompany the specimen with a more detailed report on the character and treatment of the diptheritic affection as observed in his practice, at the next meeting.

The following resolutions were next offered by Dr. Wickersham, approving of the establishment of a College of Pharmacy in our city:

*Whereas*, As we learn that there has been a College of Pharmacy established in this city, where apothecaries may be taught to distinguish between pure and impure medicines, and the improved methods of compounding them, Therefore,

*Resolved*, That this movement receives our warmest approbation, and as we feel ourselves directly interested in its success, we will regret if anything should arise to dim its lustre, or to render it less efficient than its most sanguine supporters believe it will be.

*Resolved*, That as the experience of every medical man has proved that we have in our midst many so-called pharmaceutists who are inefficient and incompetent to perform the important duties of their calling, we will in our professional capacity have additional confidence in those who avail themselves of the opportunity that now offers to make themselves adepts in their profession.

Dr. Andrews invited the Society to attend the microscopic entertainment of the Academy of Sciences, on tuesday evening next. Also an invitation was read to attend the Introductory Lecture before the College of Pharmacy, by Prof. Rauch, M. D.

Dr. Allport extended an invitation to the Society to meet at his residence the next regular evening. On motion, the invitation was cheerfully accepted, and the Society adjourned.

E. A. STEELE, *Sec'y pro tem.*

